# Female dispersion and sex ratios interact in the evolution of mating behavior: a computational model

**DOI:** 10.1038/s41598-018-20790-7

**Published:** 2018-02-06

**Authors:** B. V. Gomes, D. M. Guimarães, D. Szczupak, K. Neves

**Affiliations:** Morphological Sciences Graduate Program, Institute of Biomedical Sciences, Federal University of Rio de Janeiro, Rio de Janeiro, Brazil

## Abstract

The evolution of mating strategies is not well understood. Several hypotheses have been proposed to explain the variation in mating strategies, with varying levels of support. Specifically, female dispersion, adult sex ratio and mate guarding have been proposed as drivers of the evolution of monogamous strategies. In this study, we used an agent-based model (ABM) to examine how different mating behaviors evolve in a population under different conditions related to these putative drivers, looking to understand the interaction between them. We found an interaction among different factors in the evolution of social monogamy, and their impact is in this order: adult sex ratio (ASR), female dispersion and extra-pair copulation. Thus, when the adult sex ratio is male-biased, monogamous strategies are strongly favored. However, this is only the case if mate guarding is fully efficient, i.e., if there is no extra-pair copulation. On the other hand, in scenarios where the population is female-biased, or mate guarding is not efficient, we find that polygamous strategies are favored but proportionally to the dispersion of females. These results confirm previous findings regarding mate guarding and sex ratios, while also showing how female dispersion enters the dynamics.

## Introduction

The most recent consensus on social monogamy defines it as the sexual engagement of a male and a female for more than one breeding season^1^. This form of mating system is common in birds but very rare in mammals^2^. It is intriguing that although polygamous behavior might seem advantageous, in some cases monogamy evolves and increases individual reproductive fitness^3^. Which selective pressures lead to monogamous behavior in mammals? Why is polygamy the norm in mammals? Under which conditions should we expect monogamous or polygamous behavior to be advantageous? Over the past few decades, researchers have proposed different hypotheses to explain how social monogamy evolved for specific cases, such as in mammals^4,5^. Of these, three noteworthy hypotheses relate to male infanticide^5,6^, adult sex ratio^7,8^ and female dispersion^9,10^.

To increase reproductive fitness, males use different strategies, and one of these strategies involves the hostile behavior of slaying other males’ offspring. This strategy releases the burden of the females for caring for their offspring and makes them ready for a possible new round of copulation. The male infanticide hypothesis explains the emergence of social monogamy as a coping strategy, as the presence of a male would reduce the chances of losing its offspring. Although this is a sound hypothesis, no consensus has been reached. Studies using the same methodological approaches but with different assumptions have shown different outcomes regarding the risk of infanticide in ancestral states and monogamous behavior, both against^9,11^ and for the hypothesis^6,12^.

The female dispersion hypothesis is another interesting explanation for the emergence of social monogamy. This hypothesis states that the evolution of social monogamy is preceded by females adopting large, non-overlapping territorial ranges, either because there is no tolerance of other females or because of habitat quality and resource distribution constraining their spatial distribution^13^. The rationale is that the broad geographic dispersion of females would hinder the males’ ability to defend access to more than one female at a time, for the whole the length of a breeding season – that is, it would only be possible for one female to be guarded at a time, resulting in monogamy. Moreover, this dispersion of females would reduce the fitness of wandering males whose strategy, instead of monogamy, consists of searching for multiple females with which to mate, which means more time and resources are spent in the search^14^.

Lastly, sex ratios are important factors in the evolution of mating strategies. This framework explicitly addresses the dynamics of the numbers of active males and receptive females in a population^15^, but interestingly, it also covers emergent phenomena such as competition of males for females and mate guarding^16^, where a male remains close to his female shunning other reproductive males and avoiding female extra-pair copulation, guaranteeing paternity of her offspring. The literature on this strategy has dealt with two different measures of sex ratios that might appear interchangeable; therefore, we feel some clarification is needed. The *adult sex ratio* (ASR) is the proportion of males in the adult population that have achieved sexual maturity, and the *operational sex ratio* (OSR) is the proportion of males and females in the adult population that are apt for reproduction^17^. While ASR is stable over a breeding season, OSR depends on whether individuals are choosing to mate or not and is therefore more variable across time and not necessarily correlated with ASR^17^.

Efforts to understand the evolution of mating strategies have mainly used two experimental approaches: generalizing based on scarce ethological observations^18–20^ or reconstructing phylogenetic trees based on ancestral behaviors and inferring the causes of the evolution of social monogamy based on order of appearance^21,22^. Both approaches rely on the observation and categorization of complex behaviors, a dauntingly difficult task. Because of a lack of consensus about how these steps are to be implemented, evidence is contradictory (for an example of two contrasting views, see^6,9,11,12^).

Others have used equation-based models (EBMs) to model large-scale mechanisms and study the fitness of different mating strategies^3,23,24^. Usually, these models consider the relationships between the variables and abstract specific empirical data, except to ensure the model is realistic. These models are useful for understanding mechanisms and clarifying assumptions but are met with skepticism by ethological purists^25^. Regardless, they have clear limitations; more specifically, EBMs are more suited to study systems where local spatial dynamics are not relevant and where individual-level behavior does not substantially vary. In systems where there is local variation and heterogeneity in individual behavior, ABMs are more appropriate^26^. Agent-based modeling is a strategy that focuses on modeling individuals and their interactions instead of system-level observables and are explicitly spatially embedded. These models define the individual agents and simulate how they evolve over time, letting emergent or system-level properties appear from the bottom-up. These models allow a researcher to set specific conditions and study how particular behaviors could differently affect outcomes, allowing experimentation and more direct translation to the actual phenomena, when compared to EBMs. In recent years, this modeling approach has brought new insights to the study of ecology in general^27^ and even to the evolution of monogamy^28^.

Taking into consideration the hypotheses put forward in the literature to explain the evolution of mating behavior in mammals and to understand the interplay of factors, we aimed to understand if changes in the behavior of individuals, induced by local changes in their circumstances – external attributes, both social and ecological^29^ – could lead to the evolution of a polygamous or monogamous phenotype in the population. Specifically, we chose to investigate the female dispersion and adult sex ratio hypotheses and their consequences in competition and extra-pair copulation because these are both hypotheses that would benefit from the explicit spatial dynamics of an agent-based model (for a summary of our working hypotheses, see Table 1). In our ABM, males move around searching for potential mates. Males are either monogamous or polygamous, which determines how they search for females, and this tendency is treated as a genetic trait, which is passed to the next generation. We then follow the spread of monogamous or polygamous behaviors in the population of male agents as a primary outcome, while changing parameters of interest (see Table 2) across simulations to test our hypotheses.

**Table 1:** Working Hypotheses, behavioral outcomes and references.

Hypothesis	Behavioral Outcome	References
Female dispersion	High female dispersion favors monogamy	^9,10,38^
Adult sex ratio	Male-biased ratios favor monogamy	^7,8^
Mate guarding	Fully efficient mate-guarding favors monogamy	^19,27^

**Table 2:** Description of model parameters and simulated values.

Parameter	Values	Description
pregnancy-chance	5	The chance that a female will get pregnant in any given copulation.
number-of-males	15; 20; 25	The number of male agents in the simulation. The proportion between males is one of the main parameters we vary and they largely influence the outcome.
longevity	4	The number of breeding seasons a male agent survives - reflecting how many years males of a species are sexually active. We set it at 4 seasons, because increasing it makes the simulations much longer and more computationally costly. This parameter does not seem to qualitatively change the results, except in exacerbating whatever differences exist between the fitness of the two phenotypes.
number-of-females	20	The number of female agents in the simulation.
female-radius	5; 10; 15; 20; 25; 30; 35	This defines how much space there is between females. They are all disposed equally spaced in a rectangular grid. This is a parameter we specifically vary in every simulation, since this is of concern to the female dispersion hypothesis.
season-duration	200; 400; 600; 800	Defines how many ticks a breeding season lasts. This is grounded on the fact that males move one patch per tick. In our experiments, the total duration of the simulations was usually set at 150 generations, which makes up to 150× longevity x season-duration ticks. This is usually set at 300 ticks, but varying it has a predictable and interesting effect on the outcome.
refractory-period-duration	10; 30	How long after copulation it takes for a female to be available again. This plays a dual role in the simulation. First, it limits the rate at which a monogamous male copulates - since his monogamous female partner is always available to him. Second, it prevents the polygamous male from behaving as a monogamous one, by not allowing him to copulate repeatedly with the same female - since there’s a refractory period, he’ll necessarily go after other available females during this time.
mate-guarding	true; false	Whether monogamous males move around when their female partner is refractory; if false, it allows a large window for extra-pair copulation, essentially making mate guarding not fully efficient at all times.
percentage monogamy	outcome	The percentage of breeding season in which more than half the population of males was monogamous.

## Results

### Female dispersion hypothesis

**Figure 1:**
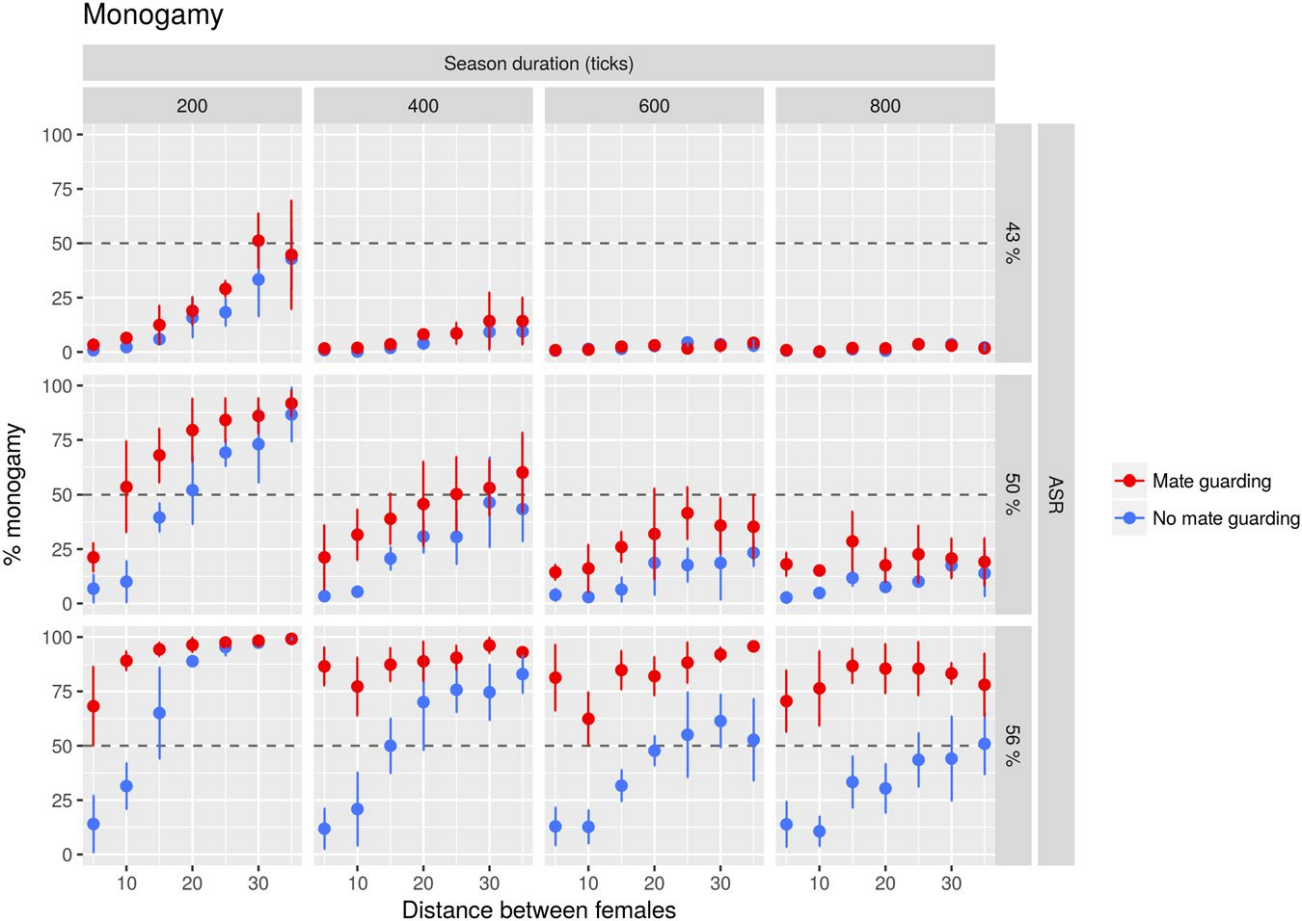
Mating strategies vary according to the interaction of ASR, female dispersion and season duration, as proposed by different hypotheses. Each point shows the mean percentage of monogamy (the percentage of seasons where over half the population of males was monogamous, see Methods Section; n = 5 replicates) and bars show 95% confidence intervals. The traced line indicates 50% of monogamy. Simulations were performed with 20 females (arbitrarily chosen), distance between females varied from 5 and 35 patches (bottom axis), breeding season varied from 200 to 800 ticks (columns), number of males shown as 15, 20 and 25 which gives ASRs of 43%, 50% and 56% (rows). Percentage monogamy (left axis) positively correlates with female distance (top left graph) and ASR (increasing from top to bottom, notice the last column) and negatively correlates with season duration (increasing from left to right, notice the first row) and the presence of mate guarding (compare blue to red data points).

Our results show that female dispersion does have an effect, in the sense predicted by the hypothesis (Fig. 1) that is, the larger the female dispersion, the more monogamous the population. However, this effect is only present in some circumstances. If the number of males is equal to or larger than that of females, there is a strong tendency toward monogamy, irrespective of the dispersion. If that situation is not the case, then if the duration of the breeding season is long, there is a strong tendency toward polygamy. When neither situation is true, female dispersion is a good predictor of monogamous behavior, as expected by the hypothesis (Fig. 1, red data points, R^2^ = 0.75, for simulations with few males (15 agents) and a short season duration (200 steps)).

We interpret these results as follows: initially, monogamous and polygamous males are equal, all of them searching for females. However, once a monogamous male finds a female, pregnancy is almost guaranteed – given a long enough season. Polygamous males, on the other hand, continue copulating after the point where most monogamous males have stopped because their female partners are pregnant. The longer the breeding season, the longer the polygamous males will continue copulating beyond the monogamous ones. For a shorter season, there is no time for polygamous males to compensate for the initial advantage of monogamous males in cases where females are very dispersed; therefore, the distance between females has a stronger effect on the fitness for each behavior. Moreover, this compensation can only occur if there are enough females available, which explains why polygamy only appears consistently in the model in situations where females outnumber males.

### Adult sex ratio and extra-pair copulation

Based on the previous results, if polygamy were to be a common mating system, we would expect that the adult sex ratio (ASR) to be female-biased in most mammals. The ASR – defined as the ratio of sexually active males, ready to mate to receptive females – is the ecological measure most closely related to the number of male and female agents as represented in our model. While to our knowledge, there is no systematic analysis of ASR in mammals, the literature shows that female-biased ASRs are not the rule^30,31^; therefore, it does not hold as a general explanation.

Another possibility is that males that cannot find females in their current location move to another location where available females can be found. However, there is another, more interesting possibility; females paired with monogamous males are not necessarily unavailable for polygamous males, because extra-pair copulation may occur^20,32^. This situation would effectively increase the number of available females at any given moment and remove the guarantees monogamous males had of a pregnancy once they find a suitable female.

To test this hypothesis, we made a simple change to the ABM; monogamous males no longer stayed beside their females – essentially making mate guarding not fully efficient – allowing the possibility of extra-pair copulation (see the Methods for details on implementation).

Allowing extra-pair copulation had the expected effect; when we decrease the monogamous male protections and allow extra-pair copulation, we see the effect of breeding season duration and female dispersion on mating behavior even when males outnumber females. The dynamics become even clearer, fitting our interpretation that monogamy is a better strategy when^1^ there are few females available relative to males^2^, when the reproductive season duration is short and^3^ when female dispersion is high.

### Regression model results

Finally, to confirm our intuition, we ran a regression of our primary outcome – the percentage of time when over half the population was monogamous – on the number of males, breeding season duration, female dispersion and a dummy variable indicating whether extra-pair copulation was present. The results show a good fit for the full model with dispersion, number of males, season duration and extra-pair copulation on the full dataset (Table 3, adjusted R² = 0.531). When we fix the parameter that is not of interest (by selecting only cases where the refractory period equals^10^), the full 4-predictor model explains the data better than models fit with each variable in isolation (full 4-predictor: adjusted R^2^ = 0.78, p < 2. 10^−16^; female dispersion: R^2^ = 0.21, p < 2. 10^−16^; season duration: R^2^ = 0.15, p < 2. 10^−16^; number of males: R^2^ = 0.36, p < 2. 10^−16^). Interestingly, the regression shows that the predictors with larger effects are the proportion of males (0.602) and female dispersion (0.310), followed by season duration and extra-pair copulation presence, which have a negative effect on monogamy (−0.288 and −0.185, respectively, all p-values < 2. 10^−16^).

**Table 3:** Summary of regression results.

	Estimate	Std. Error	t-value	p-value
*Female Spacing*	0.285	0.014	20.87	<2e-16
*Season Duration*	−0.220	0.012	−18.04	<2e-16
*Number of Males*	0.588	0.011	52.66	<2e-16
*Extra-pair Copulation*	−0.153	0.009	−16.73	<2e-16

**Multiple R-squared** 0.532.

**Adjusted R-squared** 0.531.

## Discussion

We built an agent-based model to study the interaction between the dispersion of females and the adult sex ratio in the evolution of mating systems. The main suggestion from our model is that although increased female dispersion is an important selective pressure on the evolution of polygamy, in accordance with recent literature^9^, it is overshadowed by the large effect of the adult sex ratio. In other words, polygamy is the norm in our model when either the ASR is female-biased or extra-pair copulation is widespread among females or both, and in these scenarios, the spatial distribution of females does not appear to matter. Otherwise, female dispersion is related to polygamy as predicted by the hypothesis.

Our results agree with many other findings, based on both empirical and modeling studies, that the sex ratio has a strong influence on the evolution of mating behavior. Schacht and Bell^24^ suggested that mate guarding drives the evolution of monogamy, based on results from a computational model. Similarly, using a mathematical model, Loo and colleagues^32^ concluded that efficient mate guarding is better than alternative mating strategies when populations are male-biased. Empirical studies are also in agreement with our conclusions. In humans, male-biased populations show more monogamy and lower frequency of multiple mating^8,33^. In non-human animals, studies with a crustacean, the snapping shrimp (*Alpheus angulatus*), showed that in conditions of female-biased sex ratios, males were more likely to search for new females after mating^34^.

While not described in the same terminology and framework, our model is comparable to the aforementioned models^24,32^ in that the two strategies available to male agents in our model are equivalent to the *mate guarding* and *multiple mating* strategies used in these studies. Furthermore, as interpreted, allowing extra-pair copulation in our model is equivalent to lowering the efficiency of the guarding behavior of a previously perfectly efficient mate. This situation leads to higher polygamy in the population, consistent with the findings of Loo and colleagues^32^. Lowering the efficiency of mate guarding in our model does not appear to harm the fitness of the monogamous male agent, which would lead to predictable results. When extra-pair copulation is present, the measure of monogamy does not fall uniformly but in accordance with the female dispersion theory (see the last row of graphs in Fig. 1). This suggests that in scenarios where mate guarding is not fully efficient, female dispersion overcomes the ASR as the stronger driver of monogamous behavior.

Since social monogamy is rare in mammals but appears to be explained, at least in part, by female dispersion^9^, possible conclusions from our results are that^1^ ASRs in mammals are female-biased or that^2^ mate guarding is not efficient in mammals, or equivalently, extra-pair copulation is common in mammals. This second proposition is supported in some recent studies showing that animals assumed to be genetically monogamous are only socially monogamous – this is the case of the prairie vole (*Microtus ochrogaster*), once held as a model of lifelong sexual fidelity^20^. It is also known that prairie voles that engage in extra-pair copulation have a larger range and venture outside their home territories more often^35^. Our results reinforce the case for a separation between social bonds and sexual fidelity in mammals. Moreover, our results point to sexual infidelity/mate guarding as a driver of higher/lower female availability, which in turn favors polygamous strategies, according to the dynamics in our model.

In addition to female dispersion and sex ratio, we find that another parameter, not directly related to female availability, also has an effect: the duration of the breeding season. We reason that in a short breeding season monogamous behavior is advantageous because the shorter the season, the less likely it is that a male will find more than one female, which means leaving the one female unguarded has no advantage. While the effect of breeding season duration was not expected, a longer breeding season has been implicated in the evolution of mating strategies in Phocidae^36^; however, the relationship between the length of breeding seasons and the prevalence of different mating strategies remains to be systematically investigated.

We also note that the model is not limited to the results and simulations presented here. By using a spatially embedded computational model, we can simultaneously experiment with different ecological parameters. Experimenting with different ecological parameters provides a platform to study the interaction of different factors in the evolution of mating strategies, which is otherwise difficult to do, whether in verbal models or empirical studies, due to the substantial number of elements and forces involved in the system and the impracticality of experimentally manipulating large populations. The model could be easily extended into future work to investigate the effect of factors that we did not explore in this study but which feature heavily in the literature, such as infanticide^6^ and parental care^7^, which are proposed to drive the evolution of monogamy in primates.

One possible avenue for future investigations is through experimentally controlled environments, with known space and number of individuals, in a setup such as the one used by the studies with prairie voles^20,35^, snapping shrimp^34^ and guppies^37^. With this setup, a scientist could vary the ratio of males and females, how long the animals are allowed to breed and the area available to test whether the predictions of our model empirically hold. This setup would be the live equivalent of our model, allowing the scientist to determine the magnitude of the effect of measured changes in the evolution of mating behavior. While a systematic investigation of several parameters would be unfeasible in this setup due to resource limitations, this would be an important step to check if our model predictions hold in a real-world environment.

Another way to test the consequences of our model and other models is through observational data from wild animals, which has been done before^10,38^, but the – data are hard to obtain^39^. Use of observational data would allow us to see if a combination of ASR, season duration and female density explain the variation in mating behavior as it does in our simulation. While we have tried to conduct a preliminary analysis using available data (such as the PanTHERIA database^40^;), the observations available in the database are not directly related to the measures we extracted from the model. While proxies could be used, we believe it would add too much noise, rendering the analysis uninformative. Additionally, systematic data on sexual fidelity among different species are not easily available and hard to obtain. The unavailability of data limited our analysis, but on the other hand, conclusions from this and other similar models could be used to guide a collection of data more suitable for such investigations.

In conclusion, according to our model, sex ratio is a stronger driver of the evolution of mating strategies than the spatial distribution of females, and it is even stronger when extra-pair copulation is not present or alternatively when mate guarding is fully efficient. When the population is male-biased and mate guarding is efficient, monogamous behavior is favored, otherwise, female dispersion becomes an important predictor of the mating strategies that spread in populations over generations.

## Methods

### Statistics

For the results in Table 3, percentage monogamy was regressed on female radius, season duration, number of males and a dummy variable for extra-pair copulation (1 if it was present, 0 if not). All variables were normalized to be between 0 and 1 prior to the regression. All statistical analyses and plotting were performed in R 3.4.0^41^. Raw data analyzed for the regression and figures are available in the Supplementary Data File (S1), and the R code for the regression is available in the Supplementary Data File (S3).

### Software and code availability

The agent-based model was implemented using the software NetLogo (version 5.3.1)^42^. The code is available as a Supplementary Data File (S2). For a browser-based version of the model, where you may generate different outcomes by inputting different parameters, please check the following link: https://www.dropbox.com/s/ei132u830xuurmv/Mating%20Systems%2C%20ASR%20and%20Female%20Dispersion.html?dl=0.

Upon request, all necessary data will be promptly shared.

### Spatial distribution of agents

The whole field was set as a square grid with sufficient patches to fit all females, regularly spaced. Female agents remain fixed and regularly spaced according to the female radius parameter. Male agents are dispersed randomly between the female agents at the beginning of each breeding season. We choose not to model the underlying reasons (e.g., resource inequality, see^13,23^) for the dispersion of females or variation in female behavior, since we were interested not in the cause of dispersion but in dispersion as a cause.

### Male behavioral strategy

During each breeding season, a male moves one patch per tick – the unit of time within NetLogo. The behavior of the male agent depends on whether its genotype is monogamous or polygamous. Initially, both monogamous and polygamous males search for a female, going after the one closest to their position that is *available*, i.e., a female that is neither pregnant nor refractory nor has a male closer to her. In cases where no females fulfill these criteria, the male moves to a random neighbor patch. If an available female is found, then the male will go toward her. Upon occupying the same patch, the male copulates with the female, which then either gets pregnant making her unavailable to other males until the end of the current breeding season or enters the refractory period – a short period during which the female rejects all males (even her monogamous partner).

After copulation, the behavior of monogamous and polygamous males diverges. If extra-pair copulation is not allowed (that is, mate guarding is on), monogamous males will stay beside the first female with which they copulate, effectively making her unavailable to other males. This result means that the monogamous male agent performs mate guarding to prevent extra-pair copulation (suggested by other models to be an important driver for the evolution of mating strategies; see^32^). However, guarding a mate limits the agent’s own chances of reproducing with other females it could be trying to find. Polygamous males, on the other hand, after copulating, return to their initial behavior, restarting the search for an available female.

We model males as having an underlying tendency toward monogamous or polygamous behavior. We assume this tendency to be genetic. This assumption might not be realistic, as a change in environment and social conditions (such as being raised in captivity) might change mating behavior. However, recent studies are starting to bridge the gap among mating behavior, ecological features and the underlying neurological and genetic aspects, which suggest our assumption is not one that compromises the conclusions.

### Pregnancy and births

If a female agent gets pregnant, as previously mentioned, then it stays pregnant until the next breeding season. The simulation keeps track of a gene pool (called *trait pool* in the model), consisting of the progeny of pregnant females. At the end of the breeding season, each pregnant female contributes to the gene pool, adding either a monogamous or a polygamous progeny to the gene pool. It is important to note that these will not be in the simulation immediately but only after the end of the current generation, when all the current males die and are replaced with new ones; while artificial, this is done for pragmatic reasons, to keep the male population constant and therefore the sex ratio also constant.

Whether a pregnant female will have a monogamous or polygamous male progeny will depend on the genotype of the male agent that is the father. In the model, monogamy is represented by a value of 1 and polygamy by 0. The progeny’s genotype (G_1_) is determined as follows from the genotype of the father (G_0_):

1 $${G}_{1}=round({G}_{0}+rando{m}_{normal}(\mu =0,\sigma =0.35))$$

where ***round*** is a custom rounding function – any argument larger than or equal to 0.5 is given a 1, otherwise it is given a 0 – and ***random_normal*** is a function that returns a value randomly drawn from a normal distribution with a mean and standard deviation specified by the arguments. This variation in the father’s genotype is added to prevent the population from being stuck in a specific configuration if all agents have the same genotype and were selected through trial and error to achieve sufficient variation to allow selection but not a level of variation that would add too much noise to the results. The first generation of males has an equal chance of being either monogamous or polygamous. While this genetic model is far from realistic such as it does not represent female genes, we opt for simplicity as opposed to making unwarranted assumptions about the genetics of mating behavior.

### Life cycle and breeding season

Each male agent survives for several reproductive seasons (set by a longevity parameter), after which they die. The population of males is kept constant, so that when a male dies, a new one is added to the population at a random spot in the grid, whose mating behavior is drawn randomly from the gene pool. This process means that not every pregnancy will lead to a new male with a given genotype, causing the distribution of monogamy/polygamy in the population to lag in relation to the underlying gene pool distribution. However, we see in our simulations that this does not change the results qualitatively – at the limit, for a high number of seasons, they are equivalent. The population of females is also kept constant and motionless (to keep the female density as defined by the model parameter). Since females do not vary in their traits, there is no need to renew the female agents as is done with male agents. Thus, one can assume that the number of males and females available for reproduction every breeding season does not substantially vary. While it is not the case that females lack movement or preferences, we allow only males to move based on the fact that in mammals, males are typically the ones that disperse to other groups to mate^2^ and females are typically the ones that have more choice in mating partners^43^.

We disregard the non-breeding periods. We take the model to represent only the males and females within reproductive age and only during the times where females are receptive to mating attempts by the males.

## Electronic supplementary material

Supplementary Material

Dataset S1
