# Supplementary material for: Female dispersion and sex ratios interact in the evolution of mating behavior: a computational model
Source: Sci Rep. 2018 Feb 6;8:2467. doi: 10.1038/s41598-018-20790-7 (PMC5802766; doi:10.1038/s41598-018-20790-7)
Supplement: Supplementary file 1 — Supplementary Material [file 41598_2018_20790_MOESM1_ESM.pdf]

Female dispersion and sex ratios interact in the evolution of mating behavior: a computational model

Authors: B. V. Gomes[1], D. M. Guimarães[1*], D. Szczupak[1], K. Neves[1]

Affiliations: [1] Morphological Sciences Graduate Program, Institute of Biomedical Sciences, Federal University of Rio de Janeiro, Rio de Janeiro, Brazil

*** Correspondence to: danielmgui@gmail.com**

Supplementary Material

Supplementary Figure 1. Plot of all simulations' percentage of monogamy results, colored by longevity. For a low enough longevity (2), it seems it's too few seasons for the differences in fitness to be reflected in the population and accumulate.

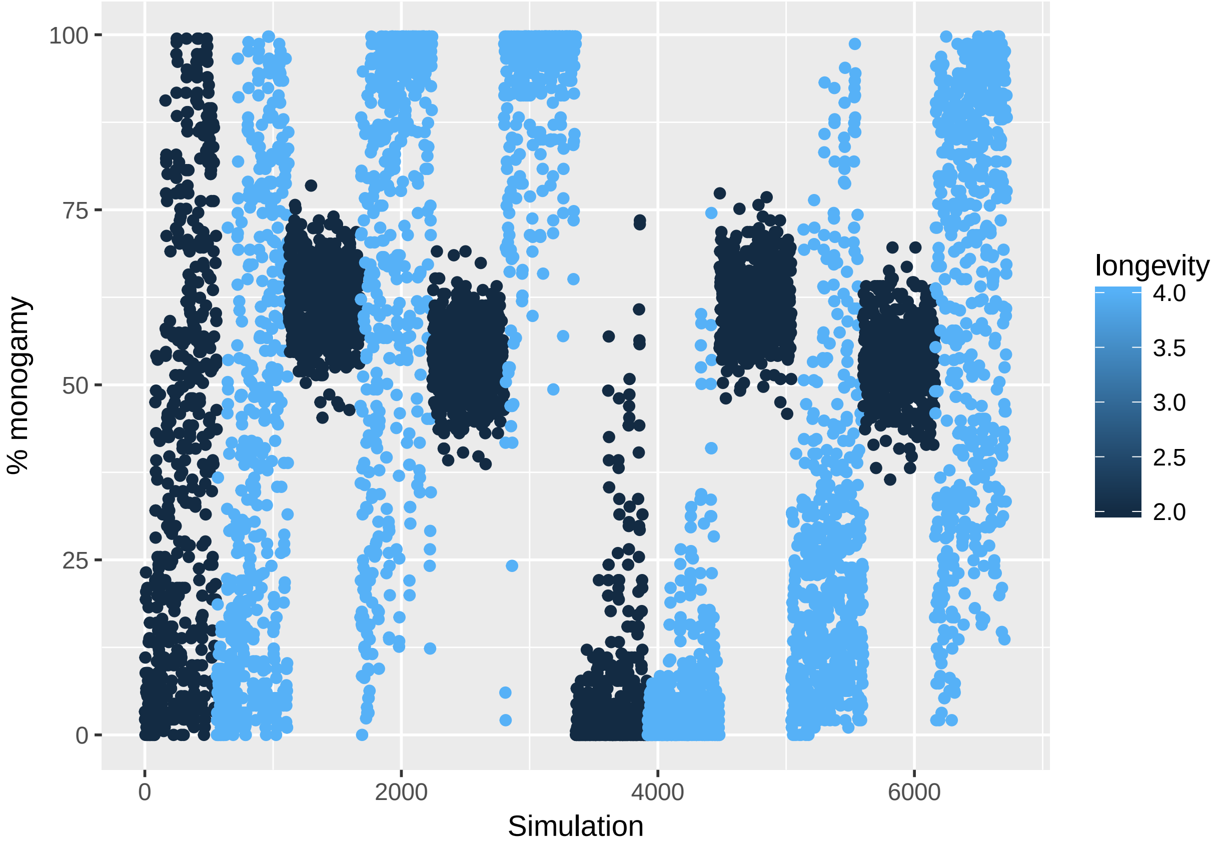

Supplementary Figure 2. Plot of simulations' percentage of monogamy, colored by pregnancy chance. A higher pregnancy chance favors polygamous behavior, but not in a absolute manner.

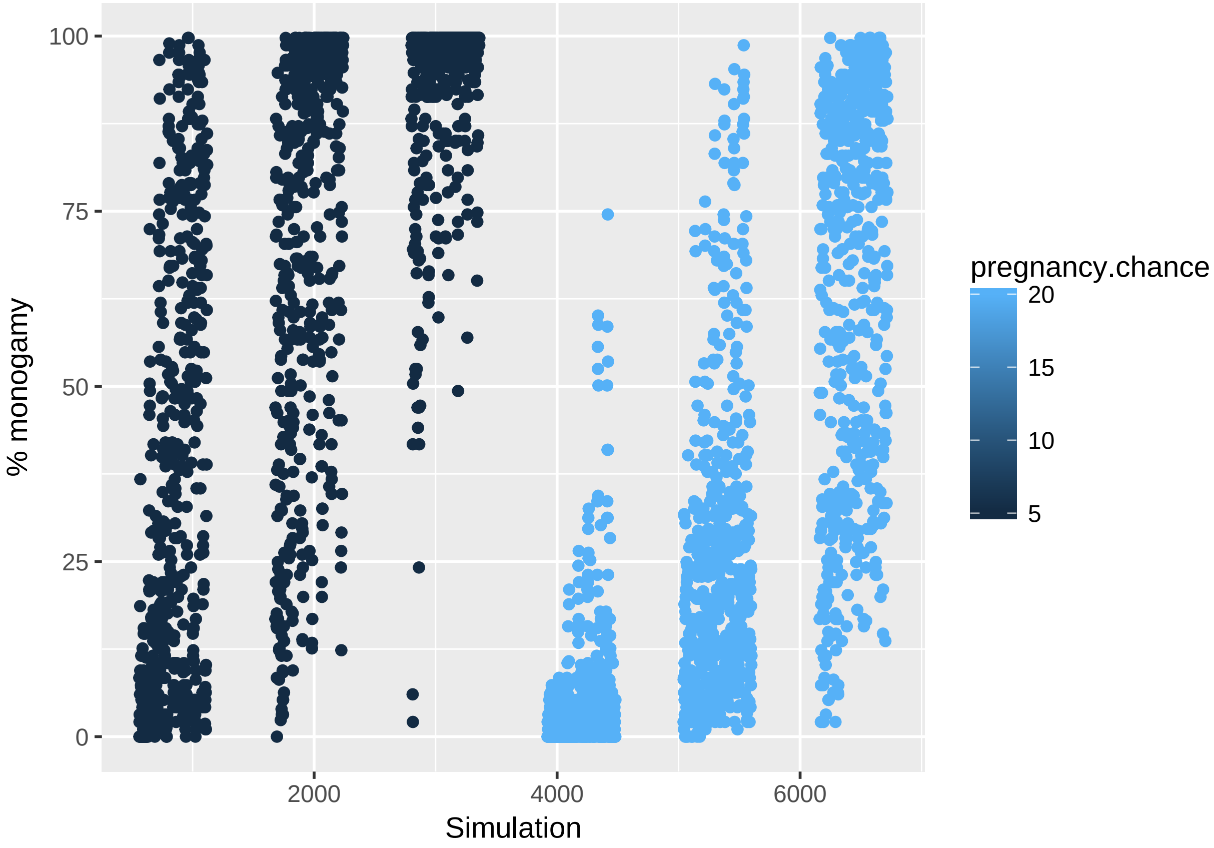

Supplementary Figure 3. Plot of simulations' percentage of monogamy, colored by whether or not mate guarding was fully efficient. The presence of extrapair copulation favors polygamous behaviors in most circumstances.

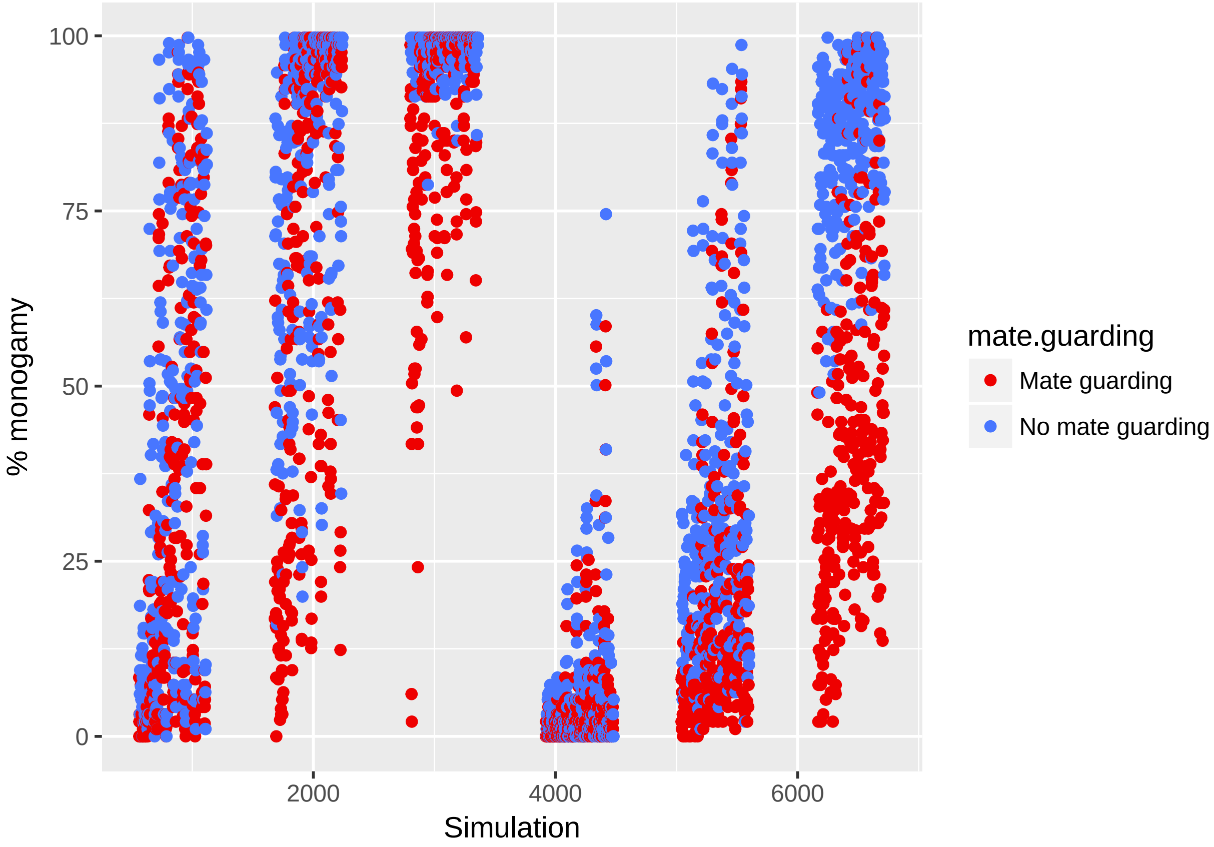

Supplementary Figure 4. Plot of simulations' percentage of monogamy, colored by number of males. A male-biased sex ratio (female number are fixed at 20 agents) limits the spread of polygamous behavior in the population over generations, especially with a low pregnancy chance.

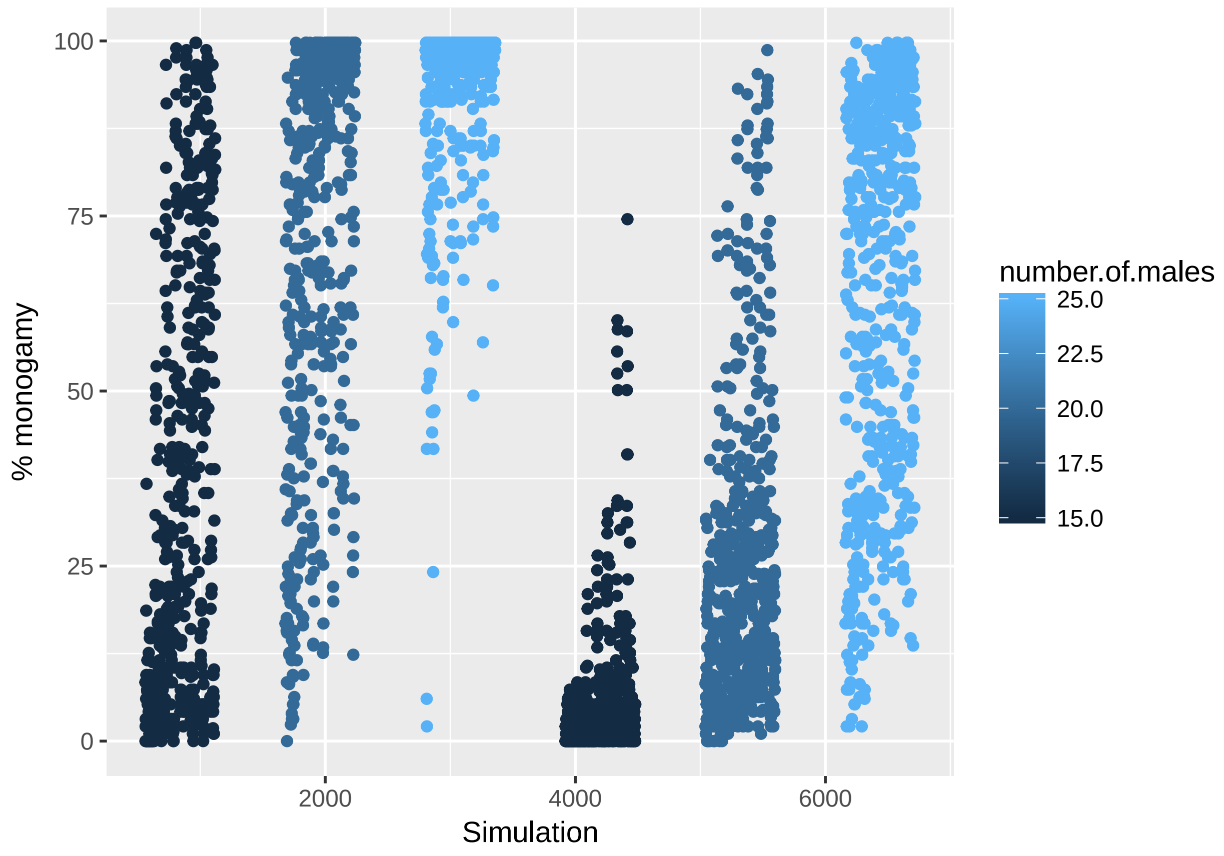

Supplementary Figure 5. Plot of simulations' percentage of monogamy, colored by breeding season duration. Although harder to interpret, visual inspection suggests higher durations favor polygamous behavior.

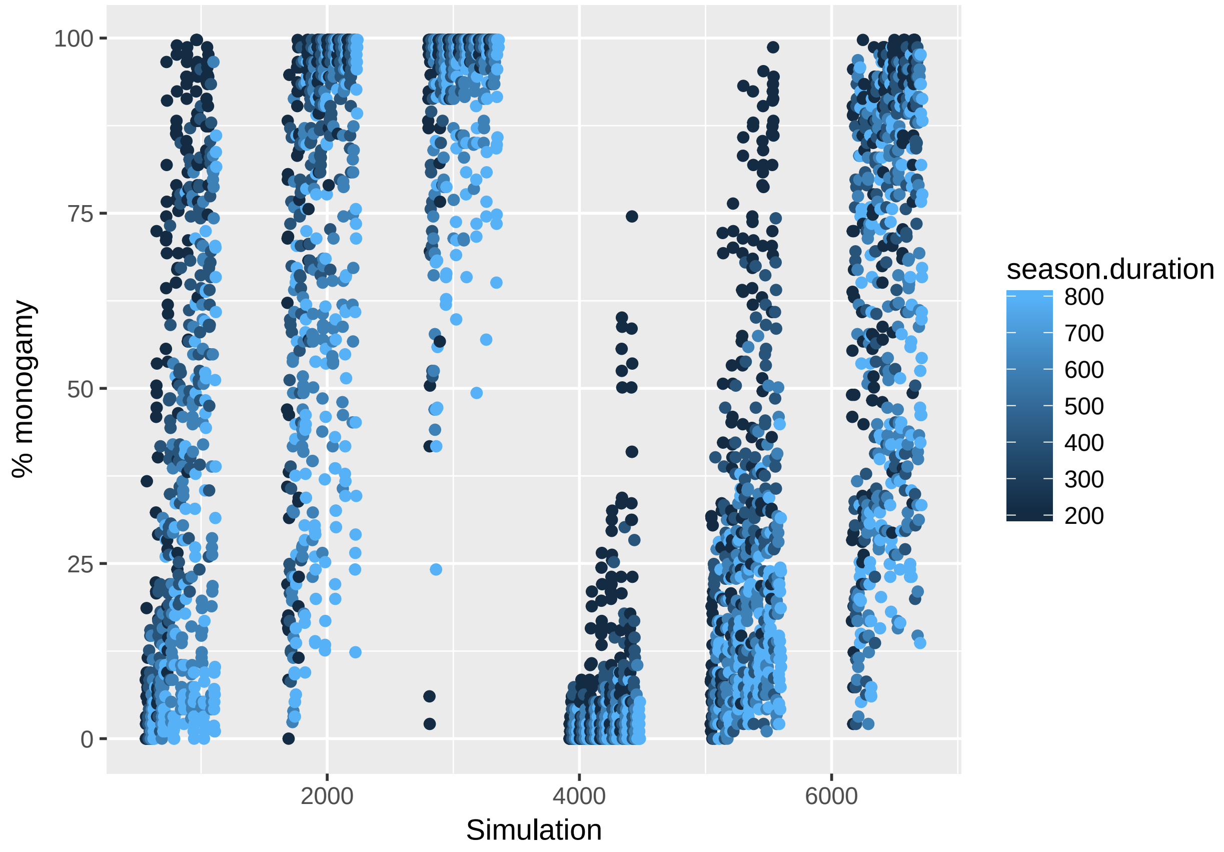

Supplementary Figure 6. Plot of simulations' percentage of monogamy, colored by refractory period. While the effect seems small and hard to observe by visual inspection, refractory period duration seems to slightly favor monogamy.

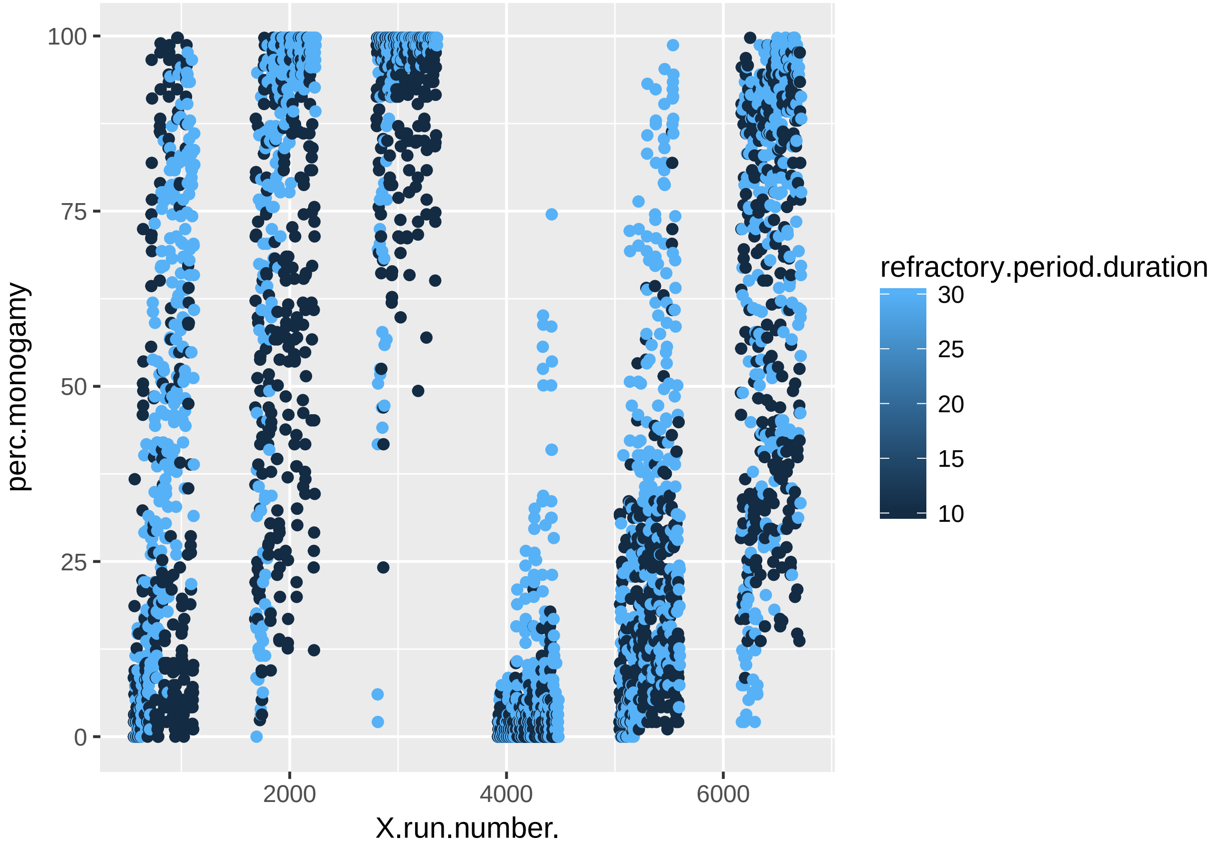

Supplementary Figure 7. Plot of simulations' percentage of monogamy as reflected on size, as a function of breeding season duration and number of males. Color reflects whether or not mate guarding was fully efficient. Notice that the size of the data points (percentage monogamy) increases from left to right - higher number of males - and from top to bottom - shorter season durations. This is another way of visualizing the main effects in our model. Also, notice that for any given row and value, blue data points tend to be smaller - inefficient of mate guarding favors polygamous behavior.

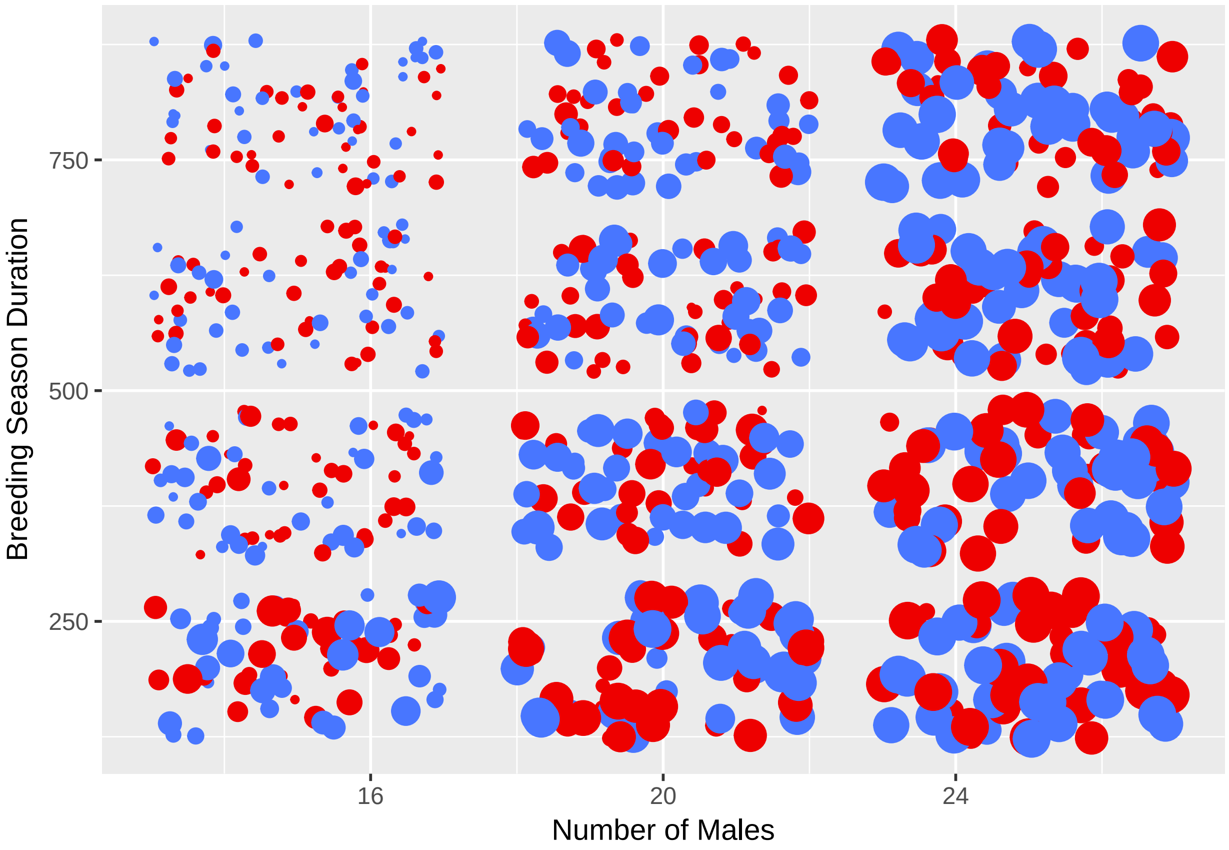

Supplementary Figure 8. Plot of simulations' percentage of monogamy as reflected on size, as a function of breeding season duration and female spacing. Color reflects whether or not mate guarding was fully efficient. Notice that the size of the data points (percentage monogamy) increases from left to right - higher female dispersion - and that the increase is much more expressive in the lower rows - where breeding seasons are shorter. Also, notice that for any given row and value, blue data points tend to be smaller - inefficient mate guarding favors polygamous behavior.

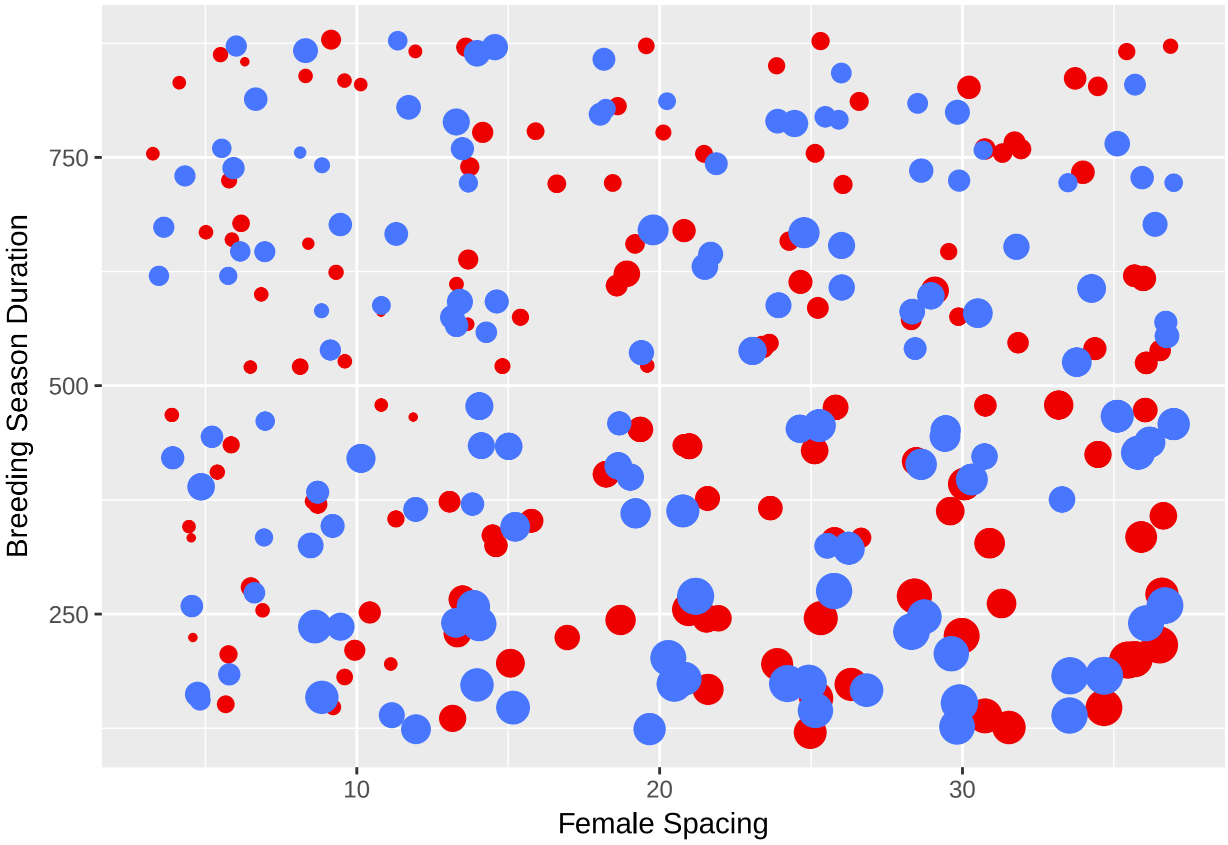

Data S2

NetLogo code for the model.

```
; defines the two breeds of turtles used
breed [males male]
breed [females female]

; variables owned by the male breed
males-own [
  monogamous ; whether or not its behavior is monogamous
  my-female ; its current selected female
  female-list ; the list of females it copulated with
]

females-own [
  my-pair ; its current male
  my-pair-monogamous ; whether or not its male is monogamous
  my-male-monogamous ; whether or not the male that impregnated her is monogamous
  gestation ; whether or not it is pregnant
  nearest-male ; the male nearest to itself
  refractory-period ; the period during which the female is unavailable after copulation
]

globals [
  trait-pool ; the list of genotypes from males who reproduced
  pregnancies ; the total number of pregnancies
  cheating ; the total number of monogamous males whose associated female copulated with an…
  total-seasons ; the number of season so far in the simulation
  monogamy-time ; the number of seasons for which, at the end, the number of monogamous mal…
  trait-monogamy-time ; the number of seasons for which, at the end, the number of monogamo…
]

to setup

  clear-all

  random-seed 10

  ; sets global variables to their starting values
  set trait-pool []
  set pregnancies 0
  set cheating 0

  set monogamy-time 0
  set trait-monogamy-time 0
  set total-seasons 0

  ; creates females, initializes their variables and position them in a grid, spaced evenly…
  let i 0
  let colnumber floor (sqrt number-of-females)
  create-females number-of-females [
    setup-female
    setxy (female-radius + (i mod colnumber) * female-radius) (female-radius + floor (i / c…
    set i i + 1
  ]

  ; creates females, initializes their variables and position them randomly among the femal…
  create-males number-of-males [
    setup-male
  ]

  ; makes patches like a chessboard, for easy visualization
  ask patches [
    set pcolor 1
    if (pxcor + pycor) mod 2 = 0 [
      set pcolor 0
    ]
  ]

  reset-ticks
```

```
end

to go

  ; updates females and males
  ask females [
    update-female
  ]

  ask males [
    update-male
  ]

  ; resets and updates everything at the end of the season
  if ticks mod season-duration = 0 [

    ; resets female variables and change the trait pool (births)
    ask females [
      if gestation > 0 [
        give-birth
      ]
      set my-male-monogamous 0
      set my-pair-monogamous 0
      set my-pair nobody
      set nearest-male nobody
      set refractory-period 0
    ]

    ; if its the end of a generation, replace males with new ones picked from the trait poo…
    if total-seasons mod longevity = 0 [
      ask males [
        replace-male 1
        die
      ]
    ]

    ; clears female list and randomize their position among the females
    ask males [
      set female-list []

      move-to one-of patches with [pxcor > min ([xcor] of females) and pycor > min ([ycor] …
      set my-female 0
    ]

  ]

  tick

end

to update-female

; check if she has a monogamous pair with any male
; if so, she's surrounded, the nearest-male is always her pair
  set my-pair-monogamous 0
  set my-pair males with [monogamous = 1 and member? myself female-list]
  ifelse any? my-pair [
    ask males with [monogamous = 1 and my-female = myself] [
      set my-female 0
    ]
    set my-pair one-of my-pair
    set my-pair-monogamous 1
    ask my-pair [
      set my-female myself
    ]
  ]
```

```
; else case: if she doesn't have a pair, then she's surrounded
; only if there's a male closer than surround radius
  [
    set my-pair nobody
    set nearest-male min-one-of males [distance myself]
    if nearest-male != nobody [
      if [distance myself] of nearest-male > 2 [
        set nearest-male nobody
      ]
    ]
  ]

  ; decreasing refractory period
  if refractory-period > 0 [
    set refractory-period refractory-period - 1
  ]

end

to find-a-female [mono]

  let available-females nobody

  ifelse mono [
    ; for a monogamous male, available females are the ones not refractory, not pregnant an…
    set available-females females with [refractory-period = 0 and gestation = 0 and (neares…
  ]
  [
    ; for a polygamous male, available females are the ones not refractory, not pregnant an…
    set available-females females with [refractory-period = 0 and gestation = 0 and (neares…
  ]

  ; picks the nearest one and follow her; if there's none, they move randomly
  ifelse any? available-females [
    set my-female min-one-of available-females [distance myself]
    follow-a-female my-female
  ] [
    move-to one-of neighbors4
  ]
end

to follow-a-female [fem]
  ; move to the closest neighbor patch to the female, unless distance to her is already 1 p…
  ifelse member? fem (females-on neighbors4) [
    move-to one-of neighbors4
  ]
  [
    move-to min-one-of neighbors4 [distance fem]
  ]
end

to copulate
  ; if the male is close enough to the female
  if member? my-female (females-on neighbors) [
    ; if there's no pregnancy
    if [gestation = 0] of my-female [
      ; adds female to list
      set female-list fput my-female female-list
      set female-list remove-duplicates female-list

      ; checks if this is cheating or not, to increase counter
      if [my-pair] of my-female != self and [my-pair] of my-female != nobody [
        set cheating cheating + 1
      ]

      ; with a small chance, the female gets pregnant
      if random 100 < pregnancy-chance [
```

```
        ask my-female [
          set my-male-monogamous [monogamous] of myself ; registered for the inheritance of…
          set gestation 1
          set pregnancies pregnancies + 1
        ]
      ]
    ]

    ; sets refractory-period
    ask my-female [
      set refractory-period refractory-period-duration
    ]

  ]

end

to update-male

  ; if monogamous
  ifelse monogamous = 1 [
    ; has a female selected
    ifelse my-female != 0 [
      ;if she is available to copulate, follow her and try to copulate
      ifelse [refractory-period <= 0] of my-female [
        follow-a-female my-female
        copulate
      ]
      [
        ; if cheating is not allowed, monogamous males will follow their female pairs at al…
        ifelse not mate-guarding [
          move-to one-of neighbors4
        ]
        [
          follow-a-female my-female
        ]
      ]
    ]
    ; if has no female selected, tries to find an available female
    [
      find-a-female true
    ]
  ]
  ; polygamous males
  [

    ; search for an available female
    find-a-female false

    ; if there's one, follow her and try to copulate
    if my-female != 0 [
      if [refractory-period <= 0] of my-female [
        copulate
      ]
    ]
  ]

end

to give-birth

  ; selects genotype: same genotype as the parent, with a small probability of deviating
  let son-monogamous my-male-monogamous + random-normal 0 0.35
  ifelse son-monogamous >= 0.5 [
    set son-monogamous 1
  ] [
    set son-monogamous 0
```

```
  ]

  ; puts genotype into trait pool
  set trait-pool fput son-monogamous trait-pool
  if length trait-pool > number-of-females * longevity [
    set trait-pool remove-item (length trait-pool - 1) trait-pool
  ]

  ; resets gestation
  set gestation 0

end

to replace-male [n-m]
  ; create a new male picking a genotype randomly from the trait pool
  hatch-males n-m [
    setup-male
    if length trait-pool > number-of-males * 2 [
      set monogamous item (random (length trait-pool)) trait-pool
    ]
  ]
end

to setup-female
  ; initializes variables
  set shape "person"
  set color pink

  set gestation 0
  set my-male-monogamous 0
  set my-pair-monogamous 0
  set my-pair nobody
  set nearest-male nobody
  set refractory-period 0

end

to setup-male
  ; initializes variables and position (random, among the females)
  set shape "person"
  set color blue

  set monogamous 1
  if random 100 > 50 [
    set monogamous 0
  ]

  set my-female 0
  set female-list []

  move-to one-of patches with [pxcor > min ([xcor] of females) and pycor > min ([ycor] of f…

end

; returns 1 if over half the population is monogamous
to-report test-half-monogamous
  let a 0
  if total-seasons > skip-seasons [
    if ticks mod season-duration = season-duration - 1 [
      set a round (count males with [monogamous = 1] / count males)
      if a = 1 [
        set monogamy-time monogamy-time + 1
      ]
    ]
  ]
  report a
end
```

```
; returns 1 if over half the trait pool is monogamous
to-report test-half-trait-monogamous
  let a 0
  if total-seasons > skip-seasons [
    if ticks mod season-duration = season-duration - 1 [
      if length trait-pool > 0 [
        set a round (sum trait-pool / length trait-pool)
        if a = 1 [
          set trait-monogamy-time trait-monogamy-time + 1
        ]
      ]
    ]
  ]
  report a
end

; reports the begginning of each season
to-report breeding-season-starts
  ifelse ticks mod season-duration = 0 [
    set total-seasons total-seasons + 1
    report 1
  ]
  [
    report 0
  ]
end

; reports the percentage of seasons in which over half the population was monogamous
to-report perc-monogamy
  ifelse total-seasons > skip-seasons [
    report monogamy-time / (total-seasons - skip-seasons) * 100
  ]
  [
    report 0
  ]
end

; reports the percentage of seasons in which over half the trait pool was monogamous
to-report perc-trait-monogamy
  ifelse total-seasons > skip-seasons [
    report trait-monogamy-time / (total-seasons - skip-seasons) * 100
  ]
  [
    report 0
  ]
end
@#$#@#$#@
GRAPHICS-WINDOW
674
10
1587
944
-1
-1
3.0
1
8
1
1
1
0
0
0
1
0
300
```

```
0
300
0
0
1
ticks
30.0

BUTTON
88
10
151
43
NIL
go
T
1
T
OBSERVER
NIL
G
NIL
NIL
1

BUTTON
155
10
224
43
go once
go
NIL
1
T
OBSERVER
NIL
NIL
NIL
NIL
1

BUTTON
12
10
85
43
NIL
setup
NIL
1
T
OBSERVER
NIL
S
NIL
NIL
1

SLIDER
17
112
228
145
number-of-females
number-of-females
1
```

```
50
20
1
1
NIL
HORIZONTAL

SLIDER
17
148
228
181
number-of-males
number-of-males
1
50
15
1
1
NIL
HORIZONTAL

SLIDER
17
199
229
232
longevity
longevity
1
20
2
1
1
seasons
HORIZONTAL

PLOT
178
495
338
615
Trait Pool
poly ------- mono
freq
0.0
2.0
0.0
10.0
true
false
"" ""
PENS
"default" 1.0 1 -16777216 true "" "histogram trait-pool"

SLIDER
231
112
443
145
female-radius
female-radius
2
40
21
1
1
```

```
NIL
HORIZONTAL

PLOT
18
495
178
615
Pregnancies
NIL
NIL
0.0
10.0
0.0
10.0
true
false
"" ""
PENS
"default" 1.0 0 -16777216 true "" "plot pregnancies"

PLOT
18
362
178
482
% Monogamy
NIL
NIL
0.0
10.0
0.0
10.0
true
false
"" ""
PENS
"default" 1.0 0 -16777216 true "" "plot 100 * count males with [monogamous = 1] / count mal…
"pen-1" 1.0 0 -7500403 true "" "plot 100 * count males with [length female-list = 1] / coun…

PLOT
178
362
338
482
Monogamy?
NIL
NIL
0.0
10.0
0.0
2.0
true
false
"" ""
PENS
"default" 1.0 0 -13791810 true "" "plot breeding-season-starts * 3"
"pen-1" 1.0 0 -16645628 true "" "plot test-half-monogamous * 2"
"pen-3" 1.0 0 -5298144 true "" "plot test-half-trait-monogamous"

PLOT
338
495
498
615
Population
poly ------- mono
```

```
freq
0.0
2.0
0.0
10.0
true
false
"" ""
PENS
"default" 1.0 1 -16777216 true "" "histogram [monogamous] of males"

PLOT
338
362
498
482
Monogamy Time
NIL
NIL
0.0
10.0
0.0
100.0
true
false
"" ""
PENS
"pen-1" 1.0 0 -16777216 true "" "plot perc-monogamy"
"pen-3" 1.0 0 -2674135 true "" "plot perc-trait-monogamy"

MONITOR
501
362
638
407
Monogamy Time
perc-monogamy
2
1
11

SLIDER
232
199
445
232
pregnancy-chance
pregnancy-chance
0
100
5
1
1
%
HORIZONTAL

TEXTBOX
1028
10
1249
68
SIMULATION VIEW
24
13.0
1

SLIDER
```

```
17
235
229
268
season-duration
season-duration
10
1000
500
10
1
ticks
HORIZONTAL

SLIDER
232
235
445
268
refractory-period-duration
refractory-period-duration
1
season-duration / 2
48
1
1
ticks
HORIZONTAL

PLOT
498
495
658
615
Extra-pair copulations
NIL
NIL
0.0
10.0
0.0
10.0
true
false
"" ""
PENS
"default" 1.0 0 -16777216 true "" "plot cheating"

SWITCH
231
148
381
181
mate-guarding
mate-guarding
0
1
-1000

TEXTBOX
17
66
167
93
Parameters
22
75.0
1
```

```
TEXTBOX
19
314
169
341
Monitors
22
43.0
1

SLIDER
450
111
663
144
skip-seasons
skip-seasons
0
50
0
5
1
NIL
HORIZONTAL

MONITOR
501
410
638
455
Trait Monogamy
perc-trait-monogamy
2
1
11

@#$#@#$#@
```

WHAT IS IT?

(a general understanding of what the model is trying to show or explain)

HOW IT WORKS

(what rules the agents use to create the overall behavior of the model)

HOW TO USE THIS MODEL

MY SECTION

(how to use the model, including a description of each of the items in the Interface tab)

THINGS TO NOTICE

(suggested things for the user to notice while running the model)

THINGS TO TRY

(suggested things for the user to try to do (move sliders, switches, etc.) with the model)

EXTENDING THE MODEL

(suggested things to add or change in the Code tab to make the model more complicated, deta…

NETLOGO FEATURES

(interesting or unusual features of NetLogo that the model uses, particularly in the Code t…

RELATED MODELS

(models in the NetLogo Models Library and elsewhere which are of related interest)

CREDITS AND REFERENCES

(a reference to the model's URL on the web if it has one, as well as any other necessary cr…
@#$#@#$#@
default
true
0
Polygon -7500403 true true 150 5 40 250 150 205 260 250

airplane
true
0
Polygon -7500403 true true 150 0 135 15 120 60 120 105 15 165 15 195 120 180 135 240 105 27…

arrow
true
0
Polygon -7500403 true true 150 0 0 150 105 150 105 293 195 293 195 150 300 150

box
false
0
Polygon -7500403 true true 150 285 285 225 285 75 150 135
Polygon -7500403 true true 150 135 15 75 150 15 285 75
Polygon -7500403 true true 15 75 15 225 150 285 150 135
Line -16777216 false 150 285 150 135
Line -16777216 false 150 135 15 75
Line -16777216 false 150 135 285 75

bug
true
0
Circle -7500403 true true 96 182 108
Circle -7500403 true true 110 127 80
Circle -7500403 true true 110 75 80
Line -7500403 true 150 100 80 30
Line -7500403 true 150 100 220 30

butterfly
true
0
Polygon -7500403 true true 150 165 209 199 225 225 225 255 195 270 165 255 150 240
Polygon -7500403 true true 150 165 89 198 75 225 75 255 105 270 135 255 150 240
Polygon -7500403 true true 139 148 100 105 55 90 25 90 10 105 10 135 25 180 40 195 85 194 1…
Polygon -7500403 true true 162 150 200 105 245 90 275 90 290 105 290 135 275 180 260 195 21…
Polygon -16777216 true false 150 255 135 225 120 150 135 120 150 105 165 120 180 150 165 225
Circle -16777216 true false 135 90 30
Line -16777216 false 150 105 195 60
Line -16777216 false 150 105 105 60

car
false
0
Polygon -7500403 true true 300 180 279 164 261 144 240 135 226 132 213 106 203 84 185 63 15…
Circle -16777216 true false 180 180 90
Circle -16777216 true false 30 180 90
Polygon -16777216 true false 162 80 132 78 134 135 209 135 194 105 189 96 180 89
Circle -7500403 true true 47 195 58
Circle -7500403 true true 195 195 58

circle
false

0
Circle -7500403 true true 0 0 300

circle 2
false
0
Circle -7500403 true true 0 0 300
Circle -16777216 true false 30 30 240

cow
false
0
Polygon -7500403 true true 200 193 197 249 179 249 177 196 166 187 140 189 93 191 78 179 72…
Polygon -7500403 true true 73 210 86 251 62 249 48 208
Polygon -7500403 true true 25 114 16 195 9 204 23 213 25 200 39 123

cylinder
false
0
Circle -7500403 true true 0 0 300

dot
false
0
Circle -7500403 true true 90 90 120

face happy
false
0
Circle -7500403 true true 8 8 285
Circle -16777216 true false 60 75 60
Circle -16777216 true false 180 75 60
Polygon -16777216 true false 150 255 90 239 62 213 47 191 67 179 90 203 109 218 150 225 192…

face neutral
false
0
Circle -7500403 true true 8 7 285
Circle -16777216 true false 60 75 60
Circle -16777216 true false 180 75 60
Rectangle -16777216 true false 60 195 240 225

face sad
false
0
Circle -7500403 true true 8 8 285
Circle -16777216 true false 60 75 60
Circle -16777216 true false 180 75 60
Polygon -16777216 true false 150 168 90 184 62 210 47 232 67 244 90 220 109 205 150 198 192…

fish
false
0
Polygon -1 true false 44 131 21 87 15 86 0 120 15 150 0 180 13 214 20 212 45 166
Polygon -1 true false 135 195 119 235 95 218 76 210 46 204 60 165
Polygon -1 true false 75 45 83 77 71 103 86 114 166 78 135 60
Polygon -7500403 true true 30 136 151 77 226 81 280 119 292 146 292 160 287 170 270 195 195…
Circle -16777216 true false 215 106 30

flag
false
0
Rectangle -7500403 true true 60 15 75 300
Polygon -7500403 true true 90 150 270 90 90 30
Line -7500403 true 75 135 90 135
Line -7500403 true 75 45 90 45

```
flower
false
0
Polygon -10899396 true false 135 120 165 165 180 210 180 240 150 300 165 300 195 240 195 19…
Circle -7500403 true true 85 132 38
Circle -7500403 true true 130 147 38
Circle -7500403 true true 192 85 38
Circle -7500403 true true 85 40 38
Circle -7500403 true true 177 40 38
Circle -7500403 true true 177 132 38
Circle -7500403 true true 70 85 38
Circle -7500403 true true 130 25 38
Circle -7500403 true true 96 51 108
Circle -16777216 true false 113 68 74
Polygon -10899396 true false 189 233 219 188 249 173 279 188 234 218
Polygon -10899396 true false 180 255 150 210 105 210 75 240 135 240

house
false
0
Rectangle -7500403 true true 45 120 255 285
Rectangle -16777216 true false 120 210 180 285
Polygon -7500403 true true 15 120 150 15 285 120
Line -16777216 false 30 120 270 120

leaf
false
0
Polygon -7500403 true true 150 210 135 195 120 210 60 210 30 195 60 180 60 165 15 135 30 12…
Polygon -7500403 true true 135 195 135 240 120 255 105 255 105 285 135 285 165 240 165 195

line
true
0
Line -7500403 true 150 0 150 300

line half
true
0
Line -7500403 true 150 0 150 150

pentagon
false
0
Polygon -7500403 true true 150 15 15 120 60 285 240 285 285 120

person
false
0
Circle -7500403 true true 110 5 80
Polygon -7500403 true true 105 90 120 195 90 285 105 300 135 300 150 225 165 300 195 300 21…
Rectangle -7500403 true true 127 79 172 94
Polygon -7500403 true true 195 90 240 150 225 180 165 105
Polygon -7500403 true true 105 90 60 150 75 180 135 105

plant
false
0
Rectangle -7500403 true true 135 90 165 300
Polygon -7500403 true true 135 255 90 210 45 195 75 255 135 285
Polygon -7500403 true true 165 255 210 210 255 195 225 255 165 285
Polygon -7500403 true true 135 180 90 135 45 120 75 180 135 210
Polygon -7500403 true true 165 180 165 210 225 180 255 120 210 135
Polygon -7500403 true true 135 105 90 60 45 45 75 105 135 135
Polygon -7500403 true true 165 105 165 135 225 105 255 45 210 60
Polygon -7500403 true true 135 90 120 45 150 15 180 45 165 90
```

```
sheep
false
15
Circle -1 true true 203 65 88
Circle -1 true true 70 65 162
Circle -1 true true 150 105 120
Polygon -7500403 true false 218 120 240 165 255 165 278 120
Circle -7500403 true false 214 72 67
Rectangle -1 true true 164 223 179 298
Polygon -1 true true 45 285 30 285 30 240 15 195 45 210
Circle -1 true true 3 83 150
Rectangle -1 true true 65 221 80 296
Polygon -1 true true 195 285 210 285 210 240 240 210 195 210
Polygon -7500403 true false 276 85 285 105 302 99 294 83
Polygon -7500403 true false 219 85 210 105 193 99 201 83

square
false
0
Rectangle -7500403 true true 30 30 270 270

square 2
false
0
Rectangle -7500403 true true 30 30 270 270
Rectangle -16777216 true false 60 60 240 240

star
false
0
Polygon -7500403 true true 151 1 185 108 298 108 207 175 242 282 151 216 59 282 94 175 3 10…

target
false
0
Circle -7500403 true true 0 0 300
Circle -16777216 true false 30 30 240
Circle -7500403 true true 60 60 180
Circle -16777216 true false 90 90 120
Circle -7500403 true true 120 120 60

tree
false
0
Circle -7500403 true true 118 3 94
Rectangle -6459832 true false 120 195 180 300
Circle -7500403 true true 65 21 108
Circle -7500403 true true 116 41 127
Circle -7500403 true true 45 90 120
Circle -7500403 true true 104 74 152

triangle
false
0
Polygon -7500403 true true 150 30 15 255 285 255

triangle 2
false
0
Polygon -7500403 true true 150 30 15 255 285 255
Polygon -16777216 true false 151 99 225 223 75 224

truck
false
0
Rectangle -7500403 true true 4 45 195 187
Polygon -7500403 true true 296 193 296 150 259 134 244 104 208 104 207 194
```

```
Rectangle -1 true false 195 60 195 105
Polygon -16777216 true false 238 112 252 141 219 141 218 112
Circle -16777216 true false 234 174 42
Rectangle -7500403 true true 181 185 214 194
Circle -16777216 true false 144 174 42
Circle -16777216 true false 24 174 42
Circle -7500403 false true 24 174 42
Circle -7500403 false true 144 174 42
Circle -7500403 false true 234 174 42

turtle
true
0
Polygon -10899396 true false 215 204 240 233 246 254 228 266 215 252 193 210
Polygon -10899396 true false 195 90 225 75 245 75 260 89 269 108 261 124 240 105 225 105 21…
Polygon -10899396 true false 105 90 75 75 55 75 40 89 31 108 39 124 60 105 75 105 90 105
Polygon -10899396 true false 132 85 134 64 107 51 108 17 150 2 192 18 192 52 169 65 172 87
Polygon -10899396 true false 85 204 60 233 54 254 72 266 85 252 107 210
Polygon -7500403 true true 119 75 179 75 209 101 224 135 220 225 175 261 128 261 81 224 74 …

wheel
false
0
Circle -7500403 true true 3 3 294
Circle -16777216 true false 30 30 240
Line -7500403 true 150 285 150 15
Line -7500403 true 15 150 285 150
Circle -7500403 true true 120 120 60
Line -7500403 true 216 40 79 269
Line -7500403 true 40 84 269 221
Line -7500403 true 40 216 269 79
Line -7500403 true 84 40 221 269

wolf
false
0
Polygon -16777216 true false 253 133 245 131 245 133
Polygon -7500403 true true 2 194 13 197 30 191 38 193 38 205 20 226 20 257 27 265 38 266 40…
Polygon -7500403 true true -1 195 14 180 36 166 40 153 53 140 82 131 134 133 159 126 188 11…

x
false
0
Polygon -7500403 true true 270 75 225 30 30 225 75 270
Polygon -7500403 true true 30 75 75 30 270 225 225 270

@#$#@#$#@
NetLogo 5.3.1
@#$#@#$#@
@#$#@#$#@
@#$#@#$#@
<experiments>
  <experiment name="Sweep" repetitions="5" runMetricsEveryStep="false">
    <setup>setup</setup>
    <go>go</go>
    <exitCondition>ticks &gt; (100 * longevity * season-duration)</exitCondition>
    <metric>monogamy-time</metric>
    <metric>trait-monogamy-time</metric>
    <metric>perc-monogamy</metric>
    <metric>perc-trait-monogamy</metric>
    <metric>total-seasons</metric>
    <metric>pregnancies</metric>
    <metric>stolen-females</metric>
    <metric>cheating</metric>
    <metric>ticks</metric>
    <enumeratedValueSet variable="pregnancy-chance">
      <value value="5"/>
```

```
      <value value="20"/>
    </enumeratedValueSet>
    <enumeratedValueSet variable="number-of-males">
      <value value="15"/>
      <value value="20"/>
      <value value="25"/>
    </enumeratedValueSet>
    <enumeratedValueSet variable="longevity">
      <value value="2"/>
      <value value="4"/>
    </enumeratedValueSet>
    <enumeratedValueSet variable="number-of-females">
      <value value="20"/>
    </enumeratedValueSet>
    <enumeratedValueSet variable="female-radius">
      <value value="5"/>
      <value value="10"/>
      <value value="15"/>
      <value value="20"/>
      <value value="25"/>
      <value value="30"/>
      <value value="35"/>
    </enumeratedValueSet>
    <enumeratedValueSet variable="season-duration">
      <value value="200"/>
      <value value="400"/>
      <value value="600"/>
      <value value="800"/>
    </enumeratedValueSet>
    <enumeratedValueSet variable="refractory-period-duration">
      <value value="10"/>
      <value value="30"/>
    </enumeratedValueSet>
    <enumeratedValueSet variable="random-parameters">
      <value value="false"/>
    </enumeratedValueSet>
    <enumeratedValueSet variable="randomize-positions">
      <value value="true"/>
    </enumeratedValueSet>
    <enumeratedValueSet variable="cheating-allowed">
      <value value="true"/>
      <value value="false"/>
    </enumeratedValueSet>
    <enumeratedValueSet variable="skip-seasons">
      <value value="20"/>
    </enumeratedValueSet>
  </experiment>
</experiments>
@#$#@#$#@
@#$#@#$#@
default
0.0
-0.2 0 0.0 1.0
0.0 1 1.0 0.0
0.2 0 0.0 1.0
link direction
true
0
Line -7500403 true 150 150 90 180
Line -7500403 true 150 150 210 180

@#$#@#$#@
0
@#$#@#$#@
```

Data S3

R code for the regression analysis.

```r
library(ggplot2)
library(broom)
library(plyr)
library(dplyr)

set.seed(10)
setwd(dirname(rstudioapi::getActiveDocumentContext()$path))

raw.data = read.csv("Data S1. Raw Data.csv",sep=";",dec=".")

normalize = function (v) {
  if (!is.numeric(v)) {
    return (v)
  }
  mx = max(v, na.rm=T)
  mn = min(v, na.rm=T)
  return ((v-mn) / (mx-mn))
}

wdata = raw.data %>% mutate(n.monogamy = normalize(perc.monogamy),
                            n.radius = normalize(female.radius),
                            n.season = normalize(season.duration),
                            n.males = normalize(number.of.males),
                            )

m = lm(data = wdata, n.monogamy ~ n.radius + n.season + n.males + mate.guarding)
summary(m)
```
